# Supplementary material for: The impact of preferential trade agreements on bilateral trade: A structural gravity model analysis
Source: PLoS One. 2021 Mar 30;16(3):e0249118. doi: 10.1371/journal.pone.0249118 (PMC8009400; doi:10.1371/journal.pone.0249118)
Supplement: Attachment — Submitted filename: Response to Reviewers.docx [file pone.0249118.s002.docx]

**Reply to Reviewers Comments for Paper PONE-D-20-21416**

**“The impact of Regional Trade Agreements on Bilateral Trade: A Structural Gravity Model Analysis”**

**Dear Editor and Reviewers,**

We would like to commence by thanking the editor and the reviewers for their valuable time and constructive comments. Their expert knowledge of the field has helped us to strengthen the manuscript significantly. According to the valuable suggestions provided by the reviewers, we have revised the manuscript. We endeavored to address all the comments and our reflections are given below point by point.

Sincerely,

The Authors

**Response to Reviewer One’s Comments**

**Reviewer #1:**

**Reviewers' general comments:**

This article has a relatively clear structure but, in the view of the reviewer, does not comply with the Criteria for Publication 4 and 5. The language is unclear and often ambiguous. Central concepts such as, for instance, the “coverage ratio index” are also referred to as “coverage trade index”, “coverage RTAs index”, “coverage index”, etc. Acronyms are often not defined and y-axes in the figures are not labelled.

The authors state that their article makes three important contributions. First, the empirical analysis is claimed to account for the heterogeneity of RTA design by developing a RTA coverage index based on the World Trade Organization (WTO) Regional Trade Agreements Database. Unfortunately, however, the authors do neither discuss the existing literature on the heterogeneity of RTA design (e.g. Dür et al., 2014; Baccini et al., 2015; Hofmann et al., 2019) nor the alternative data sources (e.g. Dür et al., 2014; Matto et al., 2020) on RTA design. The literature review would benefit from a more elaborate discussion of these and other previous contributions. Furthermore, it would be interesting to see how the empirical results compare between the authors’ proposed coverage ratio index and the existing indicators of RTA design (e.g. depth, flexibility, scope). At the very least, these indicators should be included as control variables in the empirical analysis. If the reviewer understands correctly, one of the main findings of the article is that RTAs which cover more industries, also generate larger increases in trade volumes. To the reviewer, this makes sense but is not a particularly intriguing finding.

According to the authors, the second contribution of this article is related to the use of the EORA input-output database and the division of trade data into intermediate trade, final goods, services trade and commodity trade. Using such fine-grained trade data is a commendable effort that deserves a more detailed discussion and interpretation throughout the article.

The authors see the third contribution of the article in the use of political distance as an instrumental variable. While the construction of this variable is discussed in the Results and Discussion section, it would be helpful for the reader to learn more about this variable in the Data and Variables section.

**Response:** Thank you very much for your valuable suggestions. In this new version, we have significantly improve the language of the entire manuscript, and believe this new version is more readable. In this new version, we have fully adopted your suggestions, especially the literature on the heterogeneity of RTAs design, which helps us enrich the literature review of this paper, so as to more clearly recognize the positioning and potential contribution of this paper in the literature. As for the explanation and elaboration of the data in this paper, this paper also makes a further supplement, especially for the calculation of the coverage index of RTAs and the instrumental variable we use, we describe it in more detail in Data and Variables section. Furthermore, we put all of our data sources in this version.

**Specific comments:**

1: Figure 1 is missing y-axis labels. It is not obvious to the reviewer, what the first y-axis shows and what the second y-axis shows. Page 3.

**Response:** Thank you for the valuable comments and suggestions. We give a more detailed description of Figure 1.

|  | good notifications | Services notifications | Accessions to an RTA | Cumulative Notifications of RTAs in force | Cumulative Number of RTAs in force |
| --- | --- | --- | --- | --- | --- |
| 1989 | 1 | 1 | 0 | 28 | 22 |
| 1990 | 0 | 0 | 0 | 28 | 22 |
| 1991 | 3 | 0 | 0 | 31 | 25 |
| 1992 | 2 | 0 | 0 | 33 | 27 |
| 1993 | 6 | 0 | 0 | 39 | 33 |
| 1994 | 5 | 2 | 0 | 46 | 39 |
| 1995 | 8 | 1 | 2 | 57 | 47 |
| 1996 | 8 | 0 | 0 | 65 | 55 |
| 1997 | 6 | 1 | 0 | 72 | 61 |
| 1998 | 6 | 0 | 0 | 78 | 67 |
| 1999 | 5 | 1 | 2 | 86 | 72 |
| 2000 | 10 | 1 | 0 | 97 | 82 |
| 2001 | 9 | 4 | 0 | 110 | 91 |
| 2002 | 9 | 5 | 1 | 125 | 100 |
| 2003 | 15 | 6 | 0 | 146 | 114 |
| 2004 | 10 | 7 | 2 | 165 | 124 |
| 2005 | 13 | 8 | 0 | 186 | 137 |
| 2006 | 17 | 10 | 0 | 213 | 154 |
| 2007 | 11 | 3 | 3 | 230 | 165 |
| 2008 | 17 | 11 | 0 | 258 | 182 |
| 2009 | 19 | 16 | 0 | 293 | 201 |
| 2010 | 15 | 9 | 0 | 317 | 214 |
| 2011 | 10 | 8 | 1 | 336 | 224 |
| 2012 | 16 | 10 | 0 | 362 | 240 |
| 2013 | 12 | 11 | 3 | 388 | 252 |
| 2014 | 12 | 10 | 0 | 410 | 264 |
| 2015 | 11 | 11 | 5 | 437 | 275 |
| 2016 | 12 | 5 | 0 | 454 | 287 |
| 2017 | 9 | 5 | 2 | 470 | 296 |
| 2018 | 3 | 4 | 0 | 477 | 300 |
| 2019 | 2 | 2 | 0 | 481 | 302 |

Figure 1 Global trend of RTAs over the years of 1989 – 2019. The y-axis on the left shows the number of different types (good notifications, services notifications and accessions to an RTA) of RTAs per year, and the y-axis on the right shows the cumulative notifications of RTAs in force and the cumulative number of RTAs in force.

2: The regional distribution of RTAs is discussed in the context of Figure 2. The authors may also consider including regional variables in their empirical analysis to control for regional patterns. Page 3.

**Response:** Thank you for the valuable comments and suggestions. In this new version, we try to add the regional variables in our empirical model (Whether a country locates in Europe or Asia). However, we already added country-pair fixed effects to control all the time-invariant country-pair factors, and the regional time-invariant variables, hence they will be omitted. The heterogenous effect of RTAs on countries from different group is necessary, therefore we used WDI database to divide country into two groups based on whether the country is with “High income”. Then we divided our sample into full groups according to whether a country is from “High income” group. The empirical results are as follows:

We further analyzed the market access effect of RTAs that could be different in terms of the economic development. We used World Development Indicators (WDI)^[1]^ to identify whether a country is High-income. Then we divided the full sample into different groups. The results of column (2) and (3) showed that the coverage index of RTAs is insignificant if the country is from high-income group. However, the depth of RTAs is still significant at 1% level. When both countries are not high-income countries, the coverage index of RTAs is significant at 1% level. We can conclude that the coverage index of RTAs can promote bilateral trade both for middle-income or low-income groups, because, to liberalize market access barriers is crucial for these countries. However, for high-income countries, the market access barriers are already less and the depth of RTAs is much important to promote bilateral trade.

Table 3

Robustness checks of the coverage index (RTA_ratio) on bilateral trade

| Variables | Full Sample  (1) | High-High  （2） | High-No High（3） | No High-No High  (4) |
| --- | --- | --- | --- | --- |
| RTA_ratio | 0.0450*** | 0.0173 | 0.0163 | 0.0849*** |
|  | (0.009) | (0.016) | (0.021) | (0.015) |
| depth_rasch | 0.0316*** | 0.0375*** | 0.0341*** | 0.0199*** |
|  | (0.004) | (0.005) | (0.010) | (0.006) |
| flexescape | -0.0031* | -0.0098*** | -0.0070 | -0.0009 |
|  | (0.002) | (0.003) | (0.004) | (0.003) |
|  | Y | Y | Y | Y |
|  | Y | Y | Y | Y |
| Obs | 721,135 | 80,252 | 166,971 | 474,912 |
| R^2^ | 0.9965 | 0.9985 | 0.9980 | 0.9959 |

Note: See note under Table 2

3: It is not clear to the reviewer why China is singled out and discussed in the context of Figure 3. From the abstract and the introduction, it appears to the reviewer that this is a general article and not specific to China or any other particular country. If China is of particular interest to the authors, they may consider explaining their case selection in more detail and including a binary variable for China RTAs in the empirical analysis. Page 3 and 4 and 14.

**Response:** Thank you for the valuable comments and suggestions. we deleted the Figure

4: The authors may consider defining the acronyms the first time they appear in the text. While most readers in the field of international trade will be familiar with the used acronyms, other scholars might not be familiar with SAFTA, AFTA and BIMSTEC etc. Page 4.

**Response:** Thank you for the valuable comments and suggestions. In this new version, we take this advice and define it the acronyms the first time they appear in the text.

It is obvious in the era of globalization that international trade is a significant way to achieve long-term sustainable development [1]. Simultaneously trade agreements are the building blocks to raise the trade-integration among the nations by removing the trade barriers [2]. Thus, developed and developing countries are liberalizing their trade system by signing bilateral and regional preferential trade agreements [3]. For example, South Asian Association for Regional Cooperation (SAARC) member has formed the Bay of Bengal Initiative for Multi-Sectoral Technical and Economic Cooperation (BIMSTEC) in 2004 to promote economic integration among the countries. In 2006, SAARC member formed their RTAs (SAFTA—South Asian Free Trade Area) and ASEAN (Association of Southeast Asian Nation) member countries established AFTA (ASEAN Free Trade Area) in 1992 to boost trade among the member countries. According to the WTO database, the numbers of regional trade agreements were 82 in 2000; while in 2019, the numbers of regional trade agreements were 302, nearly four times higher than in 2000.

5: As previously mentioned, the literature review is limited to gravity model contributions but insufficiently discusses scholarly work on RTA design and RTAs as 'Stepping Stones' vs. 'Stumbling Blocks'. Page 4.

Response: Thank you for the valuable comments and suggestions. In this new version, we added the existing literature referring to the RTA design and RTAs as 'Stepping Stones' vs. 'Stumbling Blocks'. RTA design is important and we should introduce in the Introduction section and compare our variable with the existing variables, and hence conclude our contribution clearly The contents are as follows:

This study based on the existing studies referring to the design of RTAs and the heterogeneous effect of RTAs on bilateral trade. Many scholars have constructed other variables to measure the heterogeneity of RTAs according to the content of RTAs [22-24]. The purpose of RTAs not only to create market access between members but also to establish broader economic integration rights in goods, services, and factor markets [25]. Dür et al. (2014) [22] used two different measures to operationalize the depth of RTAs. The first measure of depth is an additive index that combines seven key provisions that can be included in RTAs, and the second one relies on latent trait analysis (see Dür et al. 2014 [22]). Baccini et al. (2015) [23] measured the flexibility of RTAs and find a positive relationship between depth and flexibility for preferential trade agreements (PTAs). Hofmann et al. (2019) [24] offered a detailed assessment of preferential arrangement. They examined the coverage and legal enforceability of provisions regulating a large set of policy areas. Miroudot et al. (2010) [26] assessed RTAs through an analysis of market access and national treatment commitments at the level of 155 sub-sectors. However, they only analyzed 56 RTAs. Although some scholars have constructed the variables to measure the heterogeneity of RTAs, however, few of them explored the effect of different measure of RTAs on bilateral trade. However, we follow Miroudot et al. (2010) [26] and constructed the coverage index of RTAs by extracting all the products that are mentioned in each RTA document and match them to industry classification. We mainly focus on the effect of market access of RTAs on bilateral trade by controlling other measurements of RTAs. If an RTA covers more products from different sectors, then it will have a better market access effect. Then we analyze the coverage index of RTAs impact on bilateral trade by using the structural gravity model.

Though growing literature has focused on RTAs and their possibly welfare effects on the multilateral trading system. However, some researchers believed that bilateral free trade agreements are stepping-stones for multilateral trade liberalization, while others pointed out that bilateral trade agreements are obstacles against free trade [50]. Both believers whether the trade agreements undermine or propel the process of globalization backing their positions with logically consistent arguments. In the perspective of the obstacle [51-55] argued that RTAs acts as undermine towards multilateral growth and unleash a protectionist spiral. Further stated that RTAs effects like the spaghetti bowl effect and reduced the incentive for countries to enter into multilateral trade agreements. Regionalism, which leads to welfare losses in both member and excluded countries [56]. RTAs countries a lot oppose multilateral liberalization as they profit from the exclusion of competitors from their markets [57]. On the other side, scholars debated that RTAs acts as stepping-stones for further globalization [58-60]. In a Ministerial Meeting of the WTO in Doha (2001), governments expressed their opinion that “regional trade agreements can play an important role in promoting the liberalization and expansion of trade” [61]. Moreover, regional agreements backing the weak governments to implement reforms and make them stable even despite of domestic opposition. Partners possibly learn the advantages of liberalization once they practice limited free trade.

To sum up, both effects (RTAs stumbling blocks & stepping –stones) are empirically relevant, which can capture the complexity of the effects of regional agreements for the process of globalization. Moreover, RTAs can encourage peaceful relationships by increasing the opportunity cost of conflicts [62].

6: The authors propose a coverage ratio index (which, confusingly, they sometimes refer to as a coverage trade index, or similar versions thereof) which the reviewer understands to be equal to the share of industries covered by the RTA. If this is correct, then one of the main findings of this paper is that the more industries are covered in a RTA, the larger the effect of the RTA on bilateral trade flows. To the reviewer, this makes sense and is not necessarily surprising. The authors may consider explaining in more detail what the contribution of this finding is. Page 6.

**Response:** Thank you for the valuable comments and suggestions. In this new version, we described in more detail that how the coverage index of RTA is constructed and the reason why we use it to measure the level of market access creation of RTAs. Our method is different from the traditional depth or flexibility measurement of RTAs that try to cover the different aspects of RTAs. The effect of the coverage index of RTAs on bilateral trade is robust when we control existing variables. We further find the heterogenous effect when we consider the difference of country pair.

First, we clarify the contribution of our paper in the introduction section as follows:

This paper contributes to the RTAs and bilateral trade literature in the following ways. In contrast to previous literature, we construct the coverage index of RTAs using WTO regional trade agreements datasets to measure the level of market access of RTAs. This would be our valuable contribution to the literature as most of the study used dummies of regional trade agreements. Although some scholars measure the depth and flexibility of RTAs, few of them explore the heterogeneous effect of RTAs using different measures. We constructed the coverage index of RTAs and added other measurements in the regression, so we can explore the effect of market access on bilateral trade by controlling other variables. After that, we decompose the total trade into components and investigate the effect of the coverage of RTAs on different trade components. Specifically, this paper uses the EORA input-output database to calculate the bilateral trade flow between the agreements countries. The distinction of using the EORA database is that not only considered the intermediate goods between the national industries, but also considers the trade volume of final consumption. We divide the trade into Intermediate Trade, Final Goods Trade, Service Trade and Commodity Trade Goods to evaluate the impact of RTAs on them. Finally, we use lagged political distance as an instrumental variable to solve the endogenous issue.

Second, we put the existing variables in our regression and analysis the differences between the coverage index of RTAs and the existing variables as follows:

Many scholars have constructed other variables to measure the heterogeneity of RTAs according to the content of RTAs [22-24]. We added these variables in our model and the empirical results showd that the coverage index of RTAs has a significant positive effect on bilateral trade in full sample, which is shown in column (1) of table 3. We also find that the depth of RTAs promote bilateral trade as well, but the marginal effect (0.0316) is less than the coverage index (0.0450). Moreover, the flexibility of RTAs has negative effect on bilateral trade, and the reason behind this is that the RTAs are flexible to adjust their policies for other purposes without violating the terms of an agreement. They could not cut the tariff as they promise because they can withdraw the RTAs. This is important for countries to design flexible RTAs. Flexible RTAs provide for legally accepted opt-outs without leading to a de jure breach of an agreement and encompass exit options, duration and renegotiation clauses, reservations, escape clauses, and withdrawal clauses, and hence help members to cooperate better [23].

We further analyzed the market access effect of RTAs. We used World Development Indicators (WDI) to identify whether a country is High-income and then we divided the full sample into different groups. The result presented in column (2) - (4) of Table 3. The results of column (2) and (3) showed that the coverage index of RTAs is insignificant if the country is from high-income group. However, the depth of RTAs is still significant at 1% level. When both countries are not high-income countries, the coverage index of RTAs is significant at 1% level. Based on results we concluded that the coverage index of RTAs could promote bilateral trade both for middle-income or low-income groups, because, to liberalize market access barriers is crucial for these countries. However, for the high-income countries, the market access barriers are already less and the depth of RTAs is much more important to promote bilateral trade.

Table 3

Robustness checks of the coverage index (RTA_ratio) on bilateral trade

| Variables | Full Sample  (1) | High-High（2） | High-No High（3） | No High-No High  (4) |
| --- | --- | --- | --- | --- |
| RTA_ratio | 0.0450*** | 0.0173 | 0.0163 | 0.0849*** |
|  | (0.009) | (0.016) | (0.021) | (0.015) |
| depth_rasch | 0.0316*** | 0.0375*** | 0.0341*** | 0.0199*** |
|  | (0.004) | (0.005) | (0.010) | (0.006) |
| flexescape | -0.0031* | -0.0098*** | -0.0070 | -0.0009 |
|  | (0.002) | (0.003) | (0.004) | (0.003) |
|  | Y | Y | Y | Y |
|  | Y | Y | Y | Y |
| Obs | 721,135 | 80,252 | 166,971 | 474,912 |
| R^2^ | 0.9965 | 0.9985 | 0.9980 | 0.9959 |

Note: we divide our full sample into different samples based on whether the country is from high income group. High High denotes both countries are of high income，High Non high means one country is of high income and the other is of middle or low income. No high No high means both countries are from middle or income group

7: Figure 4 is missing a y-axis label. Page 6.

**Response:** Thank you for the valuable comments and suggestions. In this new version, we added the y-axis into previous Figure 4 and introduce the calculation in more detail. In this version, this Figure is labeled as Figure 3.

**Figure 3 The trend of coverage ratio index of RTAs over the years 2000 to 2019.** The documents of RTAs are from WTO website: <http://rtais.wto.org/UI/PublicMaintainRTAHome.aspx>. We calculate the mean value of coverage index of RTAs in each year and we can see that the tread is upward.

8: The authors may consider providing one or two examples of the economic organizations discussed on Page 7.

**Response:** Thank you for the valuable comments and suggestions. In this new version, we fully follow your advice and take two examples to describe how we deal with the coverage index of RTA between two countries.

In multilateral agreements, a country and an economic organization divided into a country and the economic organization that shares the same RTA, for example, ASEAN. For any two countries in the ASEAN, they share the same RTAs and the coverage index of RTA. In 2002, CAFTA (China and ASEAN Free Trade Area) signed by China and ASEAN countries. In this case, China and each country of ASEAN share the same RTA and the coverage index of RTA. To conclude, if an agreement is sign within an economic organization, all countries included in the economic organization would be match with each other, and finally multiple countries will share the agreement. Similarly, we treat other multilateral agreements and get the reciprocal industry penetration of agreements countries. Based on the coverage index of RTAs, this paper studies the relationship between RTAs and bilateral trade.

9: Some of the descriptive statistics in Table 1 are surprising to the reviewer. The coverage ratio index average, for instance, is 0.0379. This is far below the minimum point shown in Figure 4 (which is at around 0.2). It is also not clear to the reviewer, why the geographical distance is provided as a percentage. Page 8.

**Response:** Thank you for the valuable comments and suggestions. In this new version, we recheck our data and method and rewrite the most of the paper according to reviewers’ suggestions. The coverage index average is 0.0379 as shown in Table1, this mean value is calculated using the whole observations, because many country-pair observations don’t share a common RTA, which means that the RTA_ratio=0 for those observations. However, the trend of RTA_ratio that are shown in Figure 4 is calculated using all RTAs in force. That’s why the mean value of RTA_ratio in Table 1 is not close with Figure 4. As for the geographical distance, we take a log of the actual geographical distance, so the mean value is 8.704 (the percentage is not proper here, thank you for your kind advice).

The maximum and minimum income scale are 30.49 and 18.63 respectively. The Political distance average between trading countries is 0.793. While the mean value of the geographical distance is about 8.704. The relationship between trade and RTAs shown in Figure 5. From the figure, it is clear that RTAs and the coverage trade index have an upward trend. However, bilateral trade increase comparatively more by coverage trade index. It implies that RTAs decrease the tariff and promote trade between partner countries.

**Table 1.**

Description

| variables | definition | N | Mean | Sd | Min | Max |
| --- | --- | --- | --- | --- | --- | --- |
| lntrade | *Total Trade value* | 721,149 | 7.699 | 2.892 | 4.268 | 24.86 |
| lnmid_trade | *Intermediate goods trade value* | 721,149 | 7.350 | 2.795 | 3.930 | 24.86 |
| lnfd_trade | *final goods trade value* | 721,149 | 6.250 | 3.153 | 2.488 | 23.55 |
| lnservice | *service trade value* | 721,149 | 7.083 | 2.984 | 3.574 | 24.72 |
| lncommo | *goods trade value* | 721,149 | 6.846 | 2.764 | 3.296 | 23.59 |
| RTA_ ratio | *coverage index of RTAs* | 721,149 | 0.0379 | 0.111 | 0 | 1 |
| RTA | *RTA Dummy* | 721,149 | 0.155 | 0.362 | 0 | 1 |
| depth_rasch | *Depth of RTAs* | 721,149 | -0.027 | 0.463 | -1.433 | 2.267 |
| flexescape | *Flexibility of RTAs* | 721,149 | 0.500 | 1.276 | 0 | 4 |
| absidealdiff | *Political distance* | 721,149 | 0.793 | 0.795 | 0 | 4.887 |
| lnYi/lnYj | *Log GDP* | 721,149 | 23.81 | 2.224 | 18.63 | 30.49 |
| lnDis | *Log Distance* | 721,149 | 8.704 | 0.809 | 0.632 | 9.892 |
| Contig | *Whether contig* | 721,149 | 0.0189 | 0.136 | 0 | 1 |
| Comlang_ | *Whether common-language* | 721,149 | 0.134 | 0.341 | 0 | 1 |
| Colony | *Whether colony* | 721,149 | 0.0124 | 0.111 | 0 | 1 |
| Comcur | *Whether common currency* | 721,149 | 0.0158 | 0.125 | 0 | 1 |

10: Figure 6 is missing y-axis labels.

**Response:** Thank you for the valuable comments and suggestions. In this new version, we added y-axis. Figure 6 (in this version, it is Figure 5).

Figure 5 Basic relation between RTAs and Trade

11: The construction of the instrumental variable should be provided earlier on in the article. It is unclear to the reviewer, why a time lag of six periods in used. Page 12.

**Response:** Thank you for the valuable comments and suggestions. In this new version, we rewrite the Data and Variables section. Firstly, we describe our data and variables in more detail, especially the instrumental variable. We use a time lag of six periods. We also check a time lag of five periods and the result does not show significant difference. We revise the content as follows:

(4) We constructed an instrumental variable to deal with the potential endogenous issue. Baccini et al. (2015) [23] used democracy to measure politics and proved that the politics have significant effect on the RTAs. Bailey et al. (2017) [73] used Votes in the United Nations General Assembly (UNGA) and developed a state-of-the-art ideal point model to estimate dynamic national ideal points along a single dimension from 1946 to 2012. By Following Bailey et al. (2017) [73], we used the United Nations voting database to measure the political distance between countries^[2]^. Specifically, the database uses the distance between "ideal points" to describe the different voting options of countries in the UN General Assembly resolutions. We used a time lag of six periods of political distance as the IV for two concerns: one is that the political distance is a long-term factor and the time lag of six periods of political distance could affect a country’s decision on signing RTAs. However, the RTAs can hardly influence the time lag of six periods of political distance. Secondly, the less availability of UNGA data after 2009. We then used the time lag of six periods of political distance to use more regression observations^[3]^.

**Response to Reviewer Two’s Comments**

**Comments: Reviewer #2:**

**Reviewers' general comments:**

This paper uses a standard gravity model to analyze the effects of regional trade agreements (RTAs) on changes to bilateral trade volumes. The authors use an interesting dataset about the UN General Assembly Voting, political distance to capture the impact of RTAs on bilateral trade. The authors find that countries sharing a common RTA actually increases their trade volumes compared to those without RTAs, and they claim that there are welfare gains from signing RTAs.

Given the difficulty of the research question, I believe that the authors need to be more rigorous on how they approach the problem and clearly write down what they are finding. Most of my comments below are on the methodology and the data, and the structure of the paper.

**Response:** Thank you very much for your valuable advice. In the process of revising the paper, we have fully adopted your suggestions. As for the explanation and elaboration of the data in this paper, this paper also makes a further supplement, especially for the calculation of the coverage index of RTAs and the instrumental variable, we described it in more detail in Data and Variables section. Furthermore, we put all of our data sources in this version. The response to specific comments are as follows.

**Specific comments:**

1: In general, it is difficult to understand the main contributions of the paper. The research question is trying to understand the impact of RTAs on bilateral trade volumes. However, it is unclear to me what is really missing in the literature, and what the main contributions to the literature are. For example, calculating various trade volumes, the authors claim, is one of the main contributions. However, I do not see a good link between constructing different trade volumes and the main research question of the paper.

**Response:** Thank you for the valuable comments and suggestions. In this new version, we have significantly improve the writing of our paper, and we introduce our contribution in the Introduction section as follows:

This study based on the existing studies referring to the design of RTAs and the heterogeneous effect of RTAs on bilateral trade. Many scholars have constructed other variables to measure the heterogeneity of RTAs according to the content of RTAs [22-24]. The purpose of RTAs not only to create market access between members but also to establish broader economic integration rights in goods, services, and factor markets [25]. Dür et al. (2014) [22] used two different measures to operationalize the depth of RTAs. The first measure of depth is an additive index that combines seven key provisions that can be included in RTAs, and the second one relies on latent trait analysis (see Dür et al. 2014 [22]). Baccini et al. (2015) [23] measured the flexibility of RTAs and find a positive relationship between depth and flexibility for preferential trade agreements (PTAs). Hofmann et al. (2019) [24] offered a detailed assessment of preferential arrangement. They examined the coverage and legal enforceability of provisions regulating a large set of policy areas. Miroudot et al. (2010) [26] assessed RTAs through an analysis of market access and national treatment commitments at the level of 155 sub-sectors. However, they only analyzed 56 RTAs. Although some scholars have constructed the variables to measure the heterogeneity of RTAs, however, few of them explored the effect of different measure of RTAs on bilateral trade. However, we follow Miroudot et al. (2010) [26] and constrfucted the coverage index of RTAs by extracting all the products that are mentioned in each RTA document and match them to industry classification. We mainly focus on the effect of market access of RTAs on bilateral trade by controlling other measurements of RTAs. If an RTA covers more products from different sectors, then it will have a better market access effect. Then we analyze the coverage index of RTAs impact on bilateral trade by using the structural gravity model.

Although, we applied the structural gravity model to deal with the bias estimation. However, the endogenous issue can still occur for the sake that countries choose their RTAs partners to get more benefits. Therefore, we adopt the lagged political distance as an IV which is measured by the absolute distance between country i and country j. Subsequent ideal point estimates are based on United Nations General Assembly Voting Data [27]. For that, the 2SLS estimation is strongly robust. Our result is stable and robust after considering the endogenous issues.

This paper contributes to the RTAs and bilateral trade literature in the following ways. In contrast to previous literature, we construct the coverage index of RTAs using WTO regional trade agreements datasets to measure the level of market access of RTAs. This would be our valuable contribution to the literature as most of the study used dummies of regional trade agreements. Although some scholars measure the depth and flexibility of RTAs, few of them explore the heterogeneous effect of RTAs using different measures. We constructed the coverage index of RTAs and added other measurements in the regression, so we can explore the effect of market access on bilateral trade by controlling other variables. After that, we decompose the total trade into components and investigate the effect of the coverage of RTAs on different trade components. Specifically, this paper uses the EORA input-output database to calculate the bilateral trade flow between the agreements countries. The distinction of using the EORA database is that not only considered the intermediate goods between the national industries, but also considers the trade volume of final consumption. We divide the trade into Intermediate Trade, Final Goods Trade, Service Trade and Commodity Trade Goods to evaluate the impact of RTAs on them. Finally, we use lagged political distance as an instrumental variable to solve the endogenous issue.

2: It would be good if the authors present the data clearly. For example, it is unclear from reading the paper how the data on UN voting looks like and what kind of information it gives us. This seems to be a crucial IV that the authors test in the latter section, and it should be clearly defined and also be explained why this was chosen to be the IV instead of other potential variables.

**Response:** Thank you for the valuable comments and suggestions. In this new version, we rewrite the Data and Variables section. Firstly, we described our data and variables in more detail, especially the instrumental variable. we use a time lag of six periods. We also check a time lag of five periods and the result does not show significant difference. We revise the content as follows:

(4)	We constructed an instrumental variable to deal with the potential endogenous issue. Baccini et al. (2015) [23] used democracy to measure politics and proved that the politics have significant effect on the RTAs. Bailey et al. (2017) [73] used Votes in the United Nations General Assembly (UNGA) and developed a state-of-the-art ideal point model to estimate dynamic national ideal points along a single dimension from 1946 to 2012. By Following Bailey et al. (2017) [73], we used the United Nations voting database to measure the political distance between countries . Specifically, the database uses the distance between "ideal points" to describe the different voting options of countries in the UN General Assembly resolutions. We used a time lag of six periods of political distance as the IV for two concerns: one is that the political distance is a long-term factor and the time lag of six periods of political distance could affect a country’s decision on signing RTAs. However, the RTAs can hardly influence the time lag of six periods of political distance. Secondly, the less availability of UNGA data after 2009. We then used the time lag of six periods of political distance to use more regression observations^[4]^.

3: The authors claim that “the coverage index is more accurate in terms of the marginal trade creation effect of the trade agreement.” (line 335-line 336) I was not able to find further support for why the coverage index would be more accurate by reading the paper. Backing this claim is important because this is directly related to the main findings of the authors’ paper.

Furthermore, it would be good to show the intuition behind why the effects of RTAs (regression coefficient) jump from a coefficient of 0.15 to a coefficient of 0.57 when the authors include the RTA coverage index. Then the authors immediately conclude that (line 323-325) the RTA coverage index shows more accurate strength of RTAs. I think that there is a missing gap here, as it is not convincing why the effects more than quadruple. In fact, when the authors add country-pair fixed effects (γ_ij), the effects for RTA dummies and RTA coverage ratios become quite similar.

**Response:** Thank you for the valuable comments and suggestions. In this new version, we described the detail how the coverage index of RTA is constructed and the reason why we use it to measure the level of market access creation of RTAs. Our method is different from the traditional depth or flexibility measurement of RTAs, which try to cover the different aspects of RTAs. And the effect of the coverage index of RTAs on bilateral trade is robust when we control existing variables, and we further find heterogenous effect when we consider the difference of country pair.

Specifically, we revised our explanation of the difference between RTA dummy and the coverage index of RTAs. The dummy can only capture the average effect of RTA on bilateral trade, but cannot measure the heterogeneous effect of different RTAs, Therefore, our measurement is good. Furthermore, scholars have constructed other variables to measure the heterogeneity of RTAs, we differ from them in indicating the market access level instead of other aspects, the content is as follows:

For the first three columns, we used RTAs dummies listed in WTO. However, Baier et al. (2019) stated that dummy variables hardly capture the heterogeneous effect of RTAs (see Baier et al. 2019) [70], and hence the marginal effect is not accurate. Therefore, we constructed the coverage index of RTAs as we have mentioned above. The results of the coverage RTAs index reported in column (4)-(6) of Table 5. The results showed that the impact of the coverage index is positive and significant at 1% level. Though the direction impact is not different but the magnitude, impact is higher than the RTAs dummies (as shown in column (3) and (6)). It implies that using the coverage index to measure the market access level of RTAs, the marginal effect is larger than the average effect of the RTA dummy, and hence we can have a better understanding of the effect of RTAs to increase the bilateral trade. In the presence of coverage trade index again we find that more income more bilateral trade. However, geographical reduce bilateral trade agreements. Similarly above, others parameters promote bilateral trade. We tested the coverage index of RTAs impact on bilateral trade with economic scale holding others parameters constant. Results again validate promoting effect of coverage index on bilateral trade. The findings of the column of (4) indicated that the coverage index of RTAs has a positive impact on bilateral trade. The same currency motivates for RTAs and thus trade increases between the signed countries.

Scholars have constructed other variables to measure the heterogeneity of RTAs according to the content of RTAs [22-24]. We added these variables in our model and the empirical results showed that the coverage index of RTAs has significant positive effect on bilateral trade in full sample, which is shown in column (1) of table 3. We also find that the depth of RTAs can promote bilateral trade as well, but the marginal effect (0.0316) is less than the coverage index (0.0450). Moreover, the flexibility of RTAs has negative effect on bilateral trade, and the reason behind this is that the RTAs are flexible to adjust their policies for other purposes without violating the terms of an agreement. They could not cut the tariff as they promise because they can withdraw the RTAs. This is important for countries to design flexible RTAs. Flexible RTAs provide for legally accepted opt-outs without leading to a de jure breach of an agreement and encompass exit options, duration and renegotiation clauses, reservations, escape clauses, and withdrawal clauses, and hence help members to cooperate better [23].

We further analyzed the market access effect of RTAs. We used World Development Indicators (WDI) to identify whether a country is High-income and then we divided the full sample into different groups. The result presented in column (2) - (4) of Table 3. The results of column (2) and (3) showed that the coverage index of RTAs is insignificant if the country is from high-income group. However, the depth of RTAs is still significant at 1% level. When both countries are not high-income countries, the coverage index of RTAs is significant at 1% level. Based on results we concluded that the coverage index of RTAs could promote bilateral trade both for middle-income or low-income groups, because, to liberalize market access barriers is crucial for these countries. However, for the high-income countries, the market access barriers are already less and the depth of RTAs is much more important to promote bilateral trade.

Table 3

Robustness checks of the coverage index (RTA_ratio) on bilateral trade

| Variables | Full Sample  (1) | High-High（2） | High-NoHigh（3） | NoHigh-NoHigh  (4) |
| --- | --- | --- | --- | --- |
| RTA_ratio | 0.0450*** | 0.0173 | 0.0163 | 0.0849*** |
|  | (0.009) | (0.016) | (0.021) | (0.015) |
| depth_rasch | 0.0316*** | 0.0375*** | 0.0341*** | 0.0199*** |
|  | (0.004) | (0.005) | (0.010) | (0.006) |
| flexescape | -0.0031* | -0.0098*** | -0.0070 | -0.0009 |
|  | (0.002) | (0.003) | (0.004) | (0.003) |
|  | Y | Y | Y | Y |
|  | Y | Y | Y | Y |
| Obs | 721,135 | 80,252 | 166,971 | 474,912 |
| R^2^ | 0.9965 | 0.9985 | 0.9980 | 0.9959 |

Note: we divide our full sample into different samples based on whether the country is from high income group. High -High denotes both countries are of high income，High- No high means one country is of high income and the other is of middle or low income. No high- No high means both countries are from middle or income group

In addition, we investigate the effect of RTAs on different trade types. We decompose the bilateral trade into intermediate goods trade, final goods trade, service trade and goods trade according to the difference in the final use of traded goods and the industry to which traded goods belong. The empirical results given in Table 3. The findings of column (1) and (2) showed that RTAs with a higher degree of openness have a significant positive promotional impact on both intermediate goods trade and final goods trade. As the estimated coefficients are 0.0408 and 0.030, which means that when the coverage of the RTAs is 1, the maximum promotion effect of the implementation of the RTAs on intermediate goods trade is 4.185%. The maximum promotion effect on the final product trade is 3.045%. This means that RTAs cannot only help countries to sell final products and hence promote the welfare of countries, but also embedded in the global value chain through intermediate goods trade.

4: Understanding the content of the paper was difficult because there were frequent mechanical errors in the sentence structures. In fact, the main messages that the authors are trying to deliver are diluted as the paper is hard to comprehend.

In this new version, we have significantly improve the language of the entire manuscript, and believe this new version is more readable.

1. available at: https://datacatalog.worldbank.org/dataset/world-development-indicators
2. The data is from: http://unbisnet.un.org/
3. We also consider the time lag of five periods of political distance, and the result doesn’t change.
4. we also consider the time lag of five periods of political distance, and the result doesn’t change.
