# Supplementary material for: The impact of preferential trade agreements on bilateral trade: A structural gravity model analysis
Source: PLoS One. 2021 Mar 30;16(3):e0249118. doi: 10.1371/journal.pone.0249118 (PMC8009400; doi:10.1371/journal.pone.0249118)
Supplement: Attachment — Submitted filename: Response to Reviewers.docx [file pone.0249118.s003.docx]

**Reply to Reviewers Comments for Paper PONE-D-20-21416**

**“The impact of Regional Trade Agreements on Bilateral Trade: A Structural Gravity Model Analysis”**

**Dear Editor and Reviewers,**

We would like to commence by thanking the editor and the reviewers for their valuable time and constructive comments. Their expert knowledge of the field has helped us to strengthen the manuscript significantly. We very much appreciate Reviewer#3’s valuable suggestions, which is really helpful for us to revise the manuscript. We endeavored to address all the comments and our reflections are given below point by point.

Sincerely,

The Authors

**Response to Reviewer One’s Comments**

**Reviewer #1:**

**Reviewers' general comments:**

The reviewer very much appreciates the authors' commendable efforts to provide detailed responses to the initial comments. However, unfortunately the revised text remains difficult to follow and the research question and scholarly contribution remain largely unclear. Furthermore, the presentation of the figures and tables leaves much to be desired.

**Response:** Thank you very much for your valuable suggestions. In this new version, we have significantly improve the language of the entire manuscript, and believe this new version is more readable. In this revision, we rearrange our story and review the literature more carefully to clarify our contribution. Hopefully we can meet your demand and help you understand our contributions to existing literature.

**Response to Reviewer #3’s Comments**

**Reviewer #3:**

**Reviewers' general comments:**

Overall, I think this article has some potentially interesting contributions to make. That said, the authors need to do a better job communicating what those contributions are, illustrating the contribution empirically, and then situating them within the relevant literature(s). Moreover, the paper needs copyediting in order to make it clearer.

**Response:** We very much appreciate your valuable suggestions, which we think are helpful for us to think more deeply about this study. We are happy that you acknowledge our contribution, and in this version, we really cherish your suggestions and revise the whole paper carefully, especially the introduction section, the literature section and the product-based coverage index of PTAs section. We also delete tables and figures which are not on the core of storyline. Hopefully we can clarify our contributions in this version. Again, thank you very much for your valuable suggestions.

**Specific comments:**

1: On the whole I find the idea of constructing a product-based measure of the scope (or breadth) of PTAs and then relating this to trade flows to be a worthwhile endeavor. However, the paper needs to do a better job explaining: A) why one would want to do this, and B) what others have done in this regard. In general, I think the front end of the paper is not nearly crisp enough in this regard. You spend time talking about stumbling blocks versus stepping stones, which is not relevant at all, and much of the initial part of the paper goes over things we already know, such as the fact that PTAs have increased in number over time. I would instead have a more focused discussion at the front end of the paper that situates the current state of knowledge only with respect to PTAs/PTA design and trade flows. What have others done to assess the trade effects of PTAs and what are they potentially leaving out? Then you can offer your study as something that extends/enhances these previous studies and/or pushes it forward in unique ways.

**Response:** Thank you for the valuable comments and suggestions. We read our paper carefully and admit that the previous version does not link our contribution to existing literature well. In this version, we revise the introduction section and literature review section. And we focus on the design of PTAs and the effect of PTAs on bilateral trade. We further write in detail the difference of the product-based coverage index of PTAs and how we calculate this variable, to verify that the measure fits the reality, we take China as an example by comparing the object of the government while designing the PTAs and our measure of these PTAs, the results are reasonable. The following is the literature review section, from which hopefully we link our contribution with existing literature well.

2 Literature review

In this section, we review related literature to clarify our contribution from the literature perspective. We mainly focus on two strands of literature: the effect of PTAs on bilateral trade and the design of PTAs.

Preferential trade agreements (PTAs) have been proliferating for the last twenty years. A large literature has studied various aspects of this phenomenon and study the design of PTAs. Some researchers early pointed out that bilateral trade agreements are obstacles against free trade [34]. In the perspective of the obstacle [35-39] argued that PTAs acts as undermine towards multilateral growth and unleash a protectionist spiral. They further stated that PTAs effects like the spaghetti bowl effect and reduced the incentive for countries to enter into multilateral trade agreements. Regionalism, which leads to welfare losses in both member and excluded countries [40]. In a Ministerial Meeting of the WTO in Doha (2001), governments expressed their opinion that “regional trade agreements can play an important role in promoting the liberalization and expansion of trade” [41]. The motivation of this evolution in PTAs is not only to ease tariff barriers but also the ‘new age’ matters such as foreign direct investment, services, labor and environmental standards [28]. More scholars applied the gravity model to estimate the effect of PTAs and find a positive effect on bilateral trade [39, 40]. However, most of the studies conceptualize PTAs as a dichotomous variable, namely whether countries sign an agreement or not, and hence treat PTAs as if they were all equal in purpose while estimating the effects of PTAs. The results are fruitful and they conclude that on average PTAs can promote bilateral trade for member countries. Some scholars estimated that the long-run effect of PTAs on bilateral trade flows is 100% and the effect varies considerably across trade agreements [42, 43]. Others applied the structural gravity model to analyze the PTAs and concluded that an average treatment effect of PTAs on trade flows is 236% [44]. Dembatapitiya and Weerahewa [45] investigated the SAFTA, European Union (EU), ASEAN, BIMSTEC and NAFTA regional trade impact and found mixed results. For example, the co-efficient for EU is significant, while, SAFTA, ASEAN, BIMSTEC and North American Free Trade Agreement (NAFTA) do not show a significant impact on bilateral trade. Sampson [46] study on the evolution of China’s PTAs and stated that China’s increasing network of PTAs is important and strategic for the Asia region. Pant and Paul [47] evaluated the PTAs for India and made an argument that PTAs are sounds to be good for the intra-regional trade volume and welfare of countries as well. Besides, scholars also assess the ex-post trade effects by applying the gravity model [48, 49]. Carre`re [50] studied ex-post PTAs and claimed that intra-regional trade mostly tied to a reduction in imports from the others countries of the world. However, Moreover, regional agreements backing the weak governments to implement reforms and make them stable even despite of domestic opposition. Partners possibly learn the advantages of liberalization once they practice limited free trade.

Until recently, scholars start to investigate the content and design of PTAs [51] and study regional specifications [52] or explain functional differences in design, for example with respect to dispute settlement [53] and flexibility provisions [54]. Furthermore, they found that not only tariff cuts, but also other market access and trade-related provisions in PTAs concerning topics such as investments and intellectual property rights matter for trade flows [5], and those deep agreements usually are flexible to adjust their policies for other purposes and withdraw the PTAs without violating the terms of an agreement [6]. Besides market access of goods, many PTAs today include provisions in such trade disciplines as services, investment, standards, intellectual property, and competition rules, as well as a host of issues not directly related to trade, such as the environment. At the beginning of the study of design of PTAs, Scholars usually focus on specific PTAs and measure the strength of a wide variety of provisions in the legal texts of PTAs [10,55]. With more and more documents of PTAs are available on WTO website, scholars extend their objects and started to pay greater attention to the scope and depth of these agreements [5-10,55-58]. They focus on the broader economic integration rights in goods, services, and factor markets brought by PTAs. For example, Dür et al. [5] used two different measures to operationalize the depth of PTAs. The first measure of depth is an additive index that combines seven key provisions that can be included in PTAs, and the second one relies on latent trait analysis. Baccini et al. [6] measured the flexibility of PTAs and find a positive relationship between depth and flexibility for preferential trade agreements (PTAs). Hofmann et al. [7] offered a detailed assessment of preferential arrangement. They examined the coverage and legal enforceability of provisions regulating a large set of policy areas. In addition to the depth of PTAs, many scholars study the breadth of PTAs [59-61]. Miroudot et al. [10] tried to construct industry-based coverage index of PTAs, however, they only study 56 service PTAs. Limão claim that a broader PTA is one where partners seek to increase market access not only in product markets, but also in markets for capital, labour, and technology [59]. Based on this, Hofmann et al. construct the measure based on a count of the provisions covered by a PTA and can focus either on the 18 ‘core’ provisions [60] and recent scholars also build a measure following this method [61]. Although some scholars have constructed the variables to measure the heterogeneity of PTAs, most of them focus on the non-trade aspects of PTAs and few of them measure the coverage index from the product-based perspective, which is important under the global production network background.

In addition to the effect of PTAs on bilateral trade and the design of PTAs, this paper also investigates how PTAs affect intermediate good trade. Existing literature referring to global value chain (GVC) tend to study how deep trade agreements affect different type of trade recently [61-63]. Laget et al. [62] use trade data in both value-added and gross form to provide as complete a picture of GVC trade as possible, and find that the positive impact of deep trade agreements on GVC integration is driven by value added trade in intermediate rather than in final goods and services. Different from Laget et al. [62], scholars [61,63] use the EORA multi-regional input–output (MRIO) data to derive variables for trade in value added, as these offer greater country coverage. They found that broader PTAs have a larger impact on trade flows involving intermediates relative to flows involving all products, suggesting that GVC trade is particularly sensitive to the scope of trade policy cooperation [61]. However, they measure the coverage index of PTAs based on the number of provisions, we still don’t know whether the products mentioned in the PTAs promote the cooperation in the global production network. With the development of global production network and the shortage in the supply of some products under COVID-19 pandemic, a product-based measure for coverage of PTAs is needed to study the heterogeneity of PTAs and how this affect bilateral trade structure.

2: For me one of the major contributions is the construction of your original variable RTA_ratio. As such, I think you need to motivate with this more and make very clear what this does that is different from previous studies. What does this measure tell us that other measures have not? How is different from or similar to the DESTA depth variables? I think you could make the argument that it similar conceptually to depth but is measured in a different way (which is a unique contribution). Or, alternatively, you could make the argument that it is different in important ways to depth (which is more about the specificity of commitments related to non-trade aspects of PTAs), and this helps us better understand the effect of PTAs on trade flows. But this needs to be clear.

Then, in the empirics, you then need to compare your variable to others, such as the depth variable. For example, are these two measures correlated and, if so, how? Depending on whether you view them as complementary or overlapping this will determine, for example, whether you should include them in the same empirical models. Right now, you have them in the same econometric models later in the paper, but if they are substitute measures, they should likely not be included for conceptual and empirical reasons (multicollinearity).

**Response:** Again, thank you for the valuable comments and suggestions. This is actually the most important contribution of this paper. Although recently scholars start to pay attention to the heterogeneity of PTAs by analyzing the content of the documents. However, most of them focus on other aspects of PTAs other than the product market liberalization level. With the increasing development of global production network and the shortage of product supply under the COVID-19 pandemic, we think the product-based coverage index of PTAs are valuable to understand the heterogeneity of PTAs and their effect on bilateral trade. In this version, we rearrange the structure of our paper and add the construction of the product-based coverage index of PTAs section.

The introduction section shows the difference between this study and the previous literature:

Countries are permitted to enter into PTAs under specific conditions covering trade in goods (Article XXIV of the General Agreement on Tariffs and Trade 1994), regional or global arrangements for trade in goods between developing country members (Enabling Clause), as well as agreements covering trade in services (Article V of the General Agreement on Trade in Services). Preferential trade agreements (PTAs) are an exception to the non-discrimination principle of the WTO, because only their signatories enjoy more favourable market-access conditions. Thus, different PTAs are heterogeneous in terms of the market access level and the cooperation that goes beyond tariff reductions, in services trade, investments, standards, public procurement, competition and intellectual property rights [2-4]. Previous studies have constructed variables to measure the heterogeneity of PTAs according to the content of PTAs document [5-7]. They construct depth variable to capture cooperation in areas such as services trade, investments, standards, public procurement, competition and intellectual property rights instead of product market liberalization of PTAs and flexibility variable to measure whether the PTAs allow states to temporarily withdraw concessions. However, with global value chains (GVCs) playing increasingly significant roles in the production of goods [11] and the shortage in the supply of some products under COVID-19 pandemic [12], measuring the product coverage of PTAs and identifying the effect of the product-based market liberalization of PTAs on bilateral trade are important. Some scholars have tried to analyze schedules of commitments in services PTAs by looking at specific market access and national treatment commitments in the 155 sub-sectors of the Services Sectoral Classification List (MTN.GNS/W/120) [8-10]. However, few studies take Goods and Services together into account and construct a product-based measurement to reveal market liberalization level of PTAs.

More scholars have studied how PTAs affect bilateral trade and concluded that PTAs can influence bilateral trade through trade creation effects and diversion effects [13-16]. Trade creation effects occurs when (because of relative efficiency) domestic production of less efficient member countries displaced by the imports from more efficient member countries. While trade diversion takes place when (because of preferences), low-cost non-member country’s imports are displaced by high-cost member country’s imports. Scholars established that the trade creation (increased trade because of relative efficiency) impact could higher compared to the trade diversion effect (increased trade because of preference) [17-18]. Although some scholar’s holds an opinion that PTAs are not consistent with an important principle of the multinational trading system and leads to unfair trade practices of trade disciplines [19-24]. PTAs have become a very appealing aspect of the international trading system [25] and according to (World Bank, 2018) [26], PTAs are currently the center of many policy-makers and scholars. Broadly speaking, PTAs pave the path to achieve “deep” economic integration by eliminating tariff. As mentioned before, PTAs are flexible and the product-based coverage of PTAs may differ from each other significantly. Most of existing studies referring to the effect of PTAs on bilateral trade mostly use dummy variable to measure whether countries share PTAs [27-29], a few scholars use the depth variable and found that the depth of PTAs matters for bilateral trade [5]. When decomposing bilateral trade into different components, although scholars have investigated the effect of PTAs on intermediate trade from global value chain (GVC) perspective and found that PTAs can facilitate supply chain activity, they mainly focus on the average effect of PTAs on GVC (using dummy variable to measure PTAs) and effect of provisions related to services, investment, and competition on GVC [30-32], starting from the product-based coverage of PTAs and test its effect on different types of trade is meaningful under the global production network background. Furthermore, some scholars tried to identify the heterogeneous effect of different PTAs on trade development and find that different PTAs can have different effect on bilateral trade [33], while most studies focus on the average effect of PTAs and knowledge of effect of PTAs on trade among different types of members are not enough. To conclude, constructing the product-based coverage of PTAs and investigate the effect of the product-based coverage of PTAs on different types of bilateral trade is important and scarce in existing literature.

We add a section to introduce our product-based coverage index of PTAs:

3.2 Construction of the product-based coverage index of PTAs

We follow scholars who study the breadth of PTAs [10, 59-61] to construct a coverage index of PTAs based on the content of PTAs. We focus on the product-based coverage of PTAs instead of coverage of provisions referring to other aspects, while existing literature focus on the industry-based coverage index of service PTAs [10] or a count of provisions covered by a PTA [59-61]. Based on WTO rules on PTAs, PTAs are under specific conditions covering trade in goods (Article XXIV of the General Agreement on Tariffs and Trade 1994), regional or global arrangements for trade in goods between developing country members (Enabling Clause), as well as agreements covering trade in services (Article V of the General Agreement on Trade in Services). We firstly download all PTA documents (302 in total) manually on WTO website extract all the products whose trade barriers will be removed to promote free trade . To calculate a comparable measure for all PTAs, we match the products mentioned in each PTA document with the ISIC Rev.4, which includes 56 industries (1 to 23 are agricultural and manufacturing goods industries, 24 to 56 are service industries). We identify whether the RTA cover this industry by searching all products in goods industries and keywords in service industries. For example, China and South Korean signed a PTA which was in force in late 2015, we search the document which is in Chinese, and it mentioned products like fish, wood, bank service, etc. Based on the ISIC Rev.4, we match fish to “3. Fishing and aquaculture”, wood to “7. Manufacture of wood and of products of wood and cork, except furniture; manufacture of articles of straw and plaiting materials”, bank service to “43. Activities auxiliary to financial services and insurance activities” respectively. The coverage index of PTAs is calculated based on equation (8), where Coveredi =1 if the RTA document covers the products of industry “i”, otherwise Coveredi =0. This is similar to the count of provisions covered by a PTA [59-61], however, we differ from them by using the ratio of covered industries based on the products.

（8）

We take China as an example to verify our product-based coverage index of PTAs , which is shown in Figure 2. China's regional trade agreements since 2000 show the characteristics of differentiation: China-Switzerland free trade agreement and China-Iceland free trade agreement, which came into effect on July 1, 2014, have a broad coverage in terms of trade in goods and trade in services. Switzerland and Iceland, as the most important non EU countries in Europe, are China’s important economic and trade partners in Europe. The two agreements have wide coverage, high level of openness, and many preferential policies. They are high-quality, wide-ranging, mutually beneficial and win-win free trade agreements. They are also one of the highest level and most comprehensive free trade agreements reached by China in recent years. The product-based coverage index of this paper shows that the coverage rate of goods trade and service trade of China-Switzerland free trade agreement is 100% and nearly 90% respectively, which has a strong fit with the official data. The official statement of the China-Georgia free trade agreement states: "Georgia will immediately implement zero tariff on 96.5% of China's products, covering 99.6% of Georgia's total imports from China; China will implement zero tariff on 93.9% of Georgia's products, covering 93.8% of China's total imports from Georgia, including 90.9% of products (42.7% of total imports) immediately implementing zero tariff, and the remaining 3% of products (51.1% of total imports) The transitional period of tax reduction is five years. In the field of trade in services, the two sides have made high-quality open commitments to many service sectors. Among them, Georgia has met China's key concerns in the fields of finance, transportation, movement of natural persons, and traditional Chinese medicine services, while China has met Georgia's key concerns in the fields of tourism, shipping, and law. " The coverage index of this paper shows that the coverage rate of goods trade and service trade of China Georgia free trade agreement is 96.56% and 24.24%, which proves the accuracy of this product-based coverage index. The product-based coverage index of other PTAs referring to China shown in Figure 2 are also consistent with the description of China's regional trade agreements, which proves the accuracy of this measure.

Figure 2 the product-based coverage index of PTAs referring to China

We then show the trend of product-based coverage index of PTAs in Fig 3. It is evident from the Figure that product-based coverage index of PTAs is increasing since the 21st century. However, it is also shown in this figure that the variance of this trend is large, we calculate the mean value of product-based coverage index of all PTAs based on the year in force, this could affect the trend due to the time lag. Also, when we look deeply to the components, we find that the coverage index of service industries of PTAs is growing more significantly than the goods industries. Overall, the upward trend can be concluded if we focus on the whole picture.

**Figure 3 The trend of coverage ratio index of PTAs over the years 2000 to 2019.** The documents of PTAs are from WTO website: <http://rtais.wto.org/UI/PublicMaintainRTAHome.aspx>. We calculate the mean value of coverage index of PTAs in each year and we can see that the trend is upward.

We need to mention that in the empirical regression, we need to construct country-pair observations. In multilateral agreements, a country and an economic organization divided into a country and the economic organization that shares the same RTA, for example, ASEAN. For any two countries in the ASEAN, they share the same PTAs and the coverage index of RTA. In 2002, CAFTA (China and ASEAN Free Trade Area) signed by China and ASEAN countries. In this case, China and each country of ASEAN share the same RTA and the coverage index of RTA. To conclude, if an agreement is sign within an economic organization, all countries included in the economic organization would be match with each other, and finally multiple countries will share the agreement. Similarly, we treat other multilateral agreements and get the reciprocal industry penetration of agreements countries. Based on the coverage index of PTAs, this paper studies the relationship between PTAs and bilateral trade.

We then compare the product-based coverage index of RTAs with other depth measures, we prove that there is no multicollinearity threat:

To verify our results, we also add other measures of PTAs and compare them with the product-based coverage in our analysis. Specifically, we added the depth and flexibility variables in our empirical model to control the cooperation that goes beyond tariff reductions, in services trade, investments, standards, public procurement, competition and intellectual property rights . Dür et al. [5] used two different measures to operationalize the depth of PTAs. The first measure of depth is an additive index that combines seven key provisions that can be included in PTAs, and the second one relies on latent trait analysis (depth_rasch) [5]. Baccini et al. [6] measures the flexibility (flexescape) of PTAs based on the content and find that positive relationship between depth and flexibility holds for preferential trade agreements (PTAs). We compare the product-based coverage index of PTAs with the depth and flexibility measure and the results are shown in Table 1. We can conclude that the product-based coverage index of PTAs is positively correlated with the RTA dummy, depth of PTAs and flexibility of PTAs, and the correlation coefficients are 0.749, 0.327, 0.65 respectively, suggesting that there is no collinearity problem between PTA_ratio, depth_rasch and flexescape.

Table 1.

Correlation matrix of different measures of PTAs

|  | PTA | PTA_ratio | depth_rasch | flexescape |
| --- | --- | --- | --- | --- |
| PTA | 1.000 |  |  |  |
| PTA_ratio | 0.749*** | 1.000 |  |  |
| depth_rasch | -0.141*** | 0.327*** | 1.000 |  |
| flexescape | 0.892*** | 0.650*** | -0.093*** | 1.000 |

Note:p-values in brackets * p < 0.1, ** p < 0.05, *** p < 0.01.

3: Another contribution seems to be that you are using a different dependent variable for some of your regressions, but you don’t really sell this that effectively. If this is novel, which I believe it is, then your paper is making an important contribution that RTAs with greater scope of products covered don’t just increase trade in general they especially increase trade in intermediate goods, which are important for supply chains. This is a good contribution, but it is hidden in the manuscript.

**Response:** Thank you for the valuable comments and suggestions. With the increasing development of global production network and the shortage of product supply under the COVID-19 pandemic, the product-based coverage index of PTAs may affect bilateral trade in different ways. We follow existing literature referring to global value chain (GVC) and decompose into different components. We clarify this contribution in the introduction section, literature section, and the empirical section.

The introduction section:

When decomposing bilateral trade into different components, although scholars have investigated the effect of PTAs on intermediate trade from global value chain (GVC) perspective and found that PTAs can facilitate supply chain activity, they mainly focus on the average effect of PTAs on GVC (using dummy variable to measure PTAs) and effect of provisions related to services, investment, and competition on GVC [30-32], starting from the product-based coverage of PTAs and test its effect on different types of trade is meaningful under the global production network background. Furthermore, some scholars tried to identify the heterogeneous effect of different PTAs on trade development and find that different PTAs can have different effect on bilateral trade [33], while most studies focus on the average effect of PTAs and knowledge of effect of PTAs on trade among different types of members are not enough. To conclude, constructing the product-based coverage of PTAs and investigate the effect of the product-based coverage of PTAs on different types of bilateral trade is important and scarce in existing literature.

The literature review section:

In addition to the effect of PTAs on bilateral trade and the design of PTAs, this paper also investigates how PTAs affect intermediate good trade. Existing literature referring to global value chain (GVC) tend to study how deep trade agreements affect different type of trade recently [61-63]. Laget et al. [62] use trade data in both value-added and gross form to provide as complete a picture of GVC trade as possible, and find that the positive impact of deep trade agreements on GVC integration is driven by value added trade in intermediate rather than in final goods and services. Different from Laget et al. [62], scholars [61,63] use the EORA multi-regional input–output (MRIO) data to derive variables for trade in value added, as these offer greater country coverage. They found that broader PTAs have a larger impact on trade flows involving intermediates relative to flows involving all products, suggesting that GVC trade is particularly sensitive to the scope of trade policy cooperation [61]. However, they measure the coverage index of PTAs based on the number of provisions, we still don’t know whether the products mentioned in the PTAs promote the cooperation in the global production network. With the development of global production network and the shortage in the supply of some products under COVID-19 pandemic, a product-based measure for coverage of PTAs is needed to study the heterogeneity of PTAs and how this affect bilateral trade structure.

The empirical section:

With the increasing development of global value chains (GVCs) and the shortage in the supply of some products under COVID-19 pandemic, our measure based on the product covered in PTAs is important for studying the effect of PTAs on bilateral trade. Further, we use EORA input-output database to calculate bilateral trade volume and decompose total trade into components in terms of types of trade (intermediate goods, final goods; commodity and service trade). We investigate the product-based coverage index of PTAs on these different trade types. The empirical results given in Table 4. The findings of column (1) and (2) showed that PTAs with a higher degree of openness have a significant positive promotional impact on both intermediate goods trade and final goods trade. As the estimated coefficients are 0.0408 and 0.030, which means that when the product-based coverage of the PTAs is 1, the maximum promotion effect of the implementation of the PTAs on intermediate goods trade is 4.185%. The maximum promotion effect on the final product trade is 3.045%. PTAs cannot only help countries to sell final products and hence promote the welfare of countries, but also embedded in the global value chain through intermediate goods trade, suggesting that the product liberalization brought by PTAs can promote the development of global production network. Countries can sign trade agreements covering a wider range of products to help them better integrate into the global value chain and promote their own trade development level. Further, findings of the differential impact of PTA_ratio on service trade and final goods trade are reported in column (3) and (4) of Table 5. Results showed that regional service trade agreements have a significant promotion effect on trade flow, with coefficients of 0.0436 and 0.0411, respectively. It implies that the maximum promotion effect of the implementation of PTAs on services trade is 5.527%, and the maximum promotion effect on trade goods is 4.185%. PTAs are not only covering tariff barriers, but also the ‘new age’ matters such as foreign direct investment, services, labor and environmental standards. The service trade promotion of PTAs shows that the high-standard PTAs are helpful for countries to open up more broadly and improve the overall trade status.

Table 5

Empirical results of the coverage index (PTA_ratio) on trade components

| Variables | lnmid_trade（1） | lnfd_trade  （2） | lnservice  （3） | lncommo  （4） |
| --- | --- | --- | --- | --- |
| PTA_ratio | 0.0387*** | 0.0509*** | 0.0538*** | 0.0334*** |
|  | (0.009) | (0.010) | (0.011) | (0.008) |
| depth_rasch | 0.0313*** | 0.0263*** | 0.0420*** | 0.0153*** |
|  | (0.003) | (0.004) | (0.004) | (0.003) |
| flexescape | -0.0020 | -0.0043** | -0.0062*** | 0.0025* |
|  | (0.002) | (0.002) | (0.002) | (0.001) |
| Comcur | 0.0210** | 0.0282** | 0.0232** | -0.0036 |
|  | (0.009) | (0.011) | (0.012) | (0.007) |
| Country-pair FE | Y | Y | Y | Y |
| Country-year FE | Y | Y | Y | Y |
| Obs | 721,135 | 721,135 | 721,135 | 721,135 |
| R^2^ | 0.9966 | 0.9961 | 0.9955 | 0.9975 |

Note: See note under Table 3

4: A final contribution is the finding that perhaps the trade creating effects of wider scope PTAs are most evident in developing country dyads. This is an important contribution too, but the writing here obscures the point. Make this clear. Also, try to make the language consistent with previous efforts.

**Response:** Thank you for the valuable comments and suggestions. This is also an important contribution of this study. We divide sample into different groups based on the economic development level of member countries of PTAs, and we compare the effect of different measures on bilateral trade (we have proven that there is no muticollinearity threat in the correlation matrix), the results are interesting and we find that the trade creating effects of wider scope PTAs are most evident in developing country dyads. We revise this part as follows:

Scholars have constructed other variables to measure the heterogeneity of PTAs according to the content of PTAs [22-24]. We add these variables in our model and the empirical results show that the coverage index of PTAs has significant positive effect on bilateral trade in the full sample, which shown in column (1) of Table 3. We also find that the depth of PTAs can promote bilateral trade as well, but the marginal effect (0.0316) is less than the coverage index (0.0450). Moreover, the flexibility of PTAs has a negative effect on bilateral trade, and the reason behind this is that the PTAs are flexible to adjust their policies for other purposes without violating the terms of an agreement. They could not cut the tariff as they promise because they can withdraw the PTAs. This is important for countries to design flexible PTAs. Flexible PTAs provide for legally accepted opt-outs without leading to a de jure breach of an agreement and encompass exit options, duration and renegotiation clauses, reservations, escape clauses, and withdrawal clauses, and hence help members to cooperate better [23]. The product-based coverage of PTAs differs them in focusing on the market access openness of PTAs instead of other aspects of PTAs.

We further analyzed the market access effect of PTAs. We used World Development Indicators (WDI) to identify whether a country is High-income and then we divided the full sample into different groups. The result presented in column (2) - (4) of Table 4. The results of column (2) and (3) showed that the coverage index of PTAs is insignificant if the country is from high-income group. However, the depth of PTAs is still significant at 1% level. When both countries are not high-income countries, the coverage index of PTAs is significant at 1% level. Based on results we concluded that the product-based coverage index of PTAs could promote bilateral trade both for middle-income or low-income groups, because, to liberalize market access barriers is crucial for these countries. However, for the high-income countries, the market access barriers are already less and the depth of PTAs is much more important to promote bilateral trade. The results shed light on the globalization development for different countries. For the high-income countries, they should focus on other aspects of PTAs which can contribute to higher quality trade. However, for the less developed countries, they should liberalize the product market gradually to promote bilateral trade.

Table 4

Robustness checks of the product-based coverage index (PTA_ratio) on bilateral trade

| Variables | Full Sample  (1) | High-High  （2） | High-No High（3） | No High-No High  (4) |
| --- | --- | --- | --- | --- |
| PTA_ratio | 0.0450*** | 0.0173 | 0.0163 | 0.0849*** |
|  | (0.009) | (0.016) | (0.021) | (0.015) |
| depth_rasch | 0.0316*** | 0.0375*** | 0.0341*** | 0.0199*** |
|  | (0.004) | (0.005) | (0.010) | (0.006) |
| flexescape | -0.0031* | -0.0098*** | -0.0070 | -0.0009 |
|  | (0.002) | (0.003) | (0.004) | (0.003) |
| Country-pair FE | Y | Y | Y | Y |
| Country-year FE | Y | Y | Y | Y |
| Obs | 721,135 | 80,252 | 166,971 | 474,912 |
| R^2^ | 0.9965 | 0.9985 | 0.9980 | 0.9959 |

Note: we divide our full sample into different samples based on whether the country is high-income group. High- High denotes both countries are of high income，High- No high means one country is of high income and the other is of middle or low income. No high -No high means both countries are from middle or income group.

5: Finally, I don’t really understand the rationale for the instrumental variable regressions. What type of endogeneity are you concerned with, and how is your selected IV going to address it? You mention the inclusion of democracy and other “political” variables in other papers, but these are often used as a control variable not in a 2 stage model. Moreover, the Baccini et al (2015) paper you cite is using joint democracy: A) as a control and B) in econometric models where the dependent variable is flexibility (from DESTA), not trade volumes. Overall, if you want to keep the IV regression, much more would need to be done to explain what value added it provides and then you need to show that the IV you select satisfies the exclusion restriction requirements.

**Response:** Thank you for the valuable comments and suggestions. We totally agree with you on this and we delete the 2SLS part of this study.

6: You never really discuss what the acronym EORA stands for or explain what this data is. I had to look it up.

**Response:** Sorry that we don’t make this clear and thank you for this suggestion. We add the construction of bilateral trade section to introduce this carefully:

3.3 Construction of bilateral trade variables and other variables

Following previous studies [61-62], We use the latest release of the EORA multi-regional input–output (MIRO) tables [71]. The rows in an MRIO table indicate the use of gross output from a particular industry in a particular country and comprise two main components. The first is intermediate use, which provides information on intermediate use by both domestic industries and industries in other countries. The second is information on final demand, which is again split between demand for final goods from both domestic and foreign sources. The columns of the MRIO table provide information on the amounts of intermediates needed for the production of gross output. The column sum thus gives the sum of the domestic and foreign production of intermediates that are used in the production of output in a particular industry and country [61]. The EORA input–output tables have the distinct advantage of offering very wide country coverage when compared with other input-output databases: our sample includes 189 economies over the 1990–2015 period. We can calculate different components of total trade based on the EORA multi-regional input–output (MIRO) tables, and hence investigate the effect of product-based coverage index of PTAs on bilateral trade deeply.

7: In figure 1, what is the difference between “cumulative notifications of RTAs in force” and “cumulative number of RTAs in force”? Do you mean signed versus ratified?

**Response:** Sorry that we don’t make this clear and thank you for this suggestion. We rewrite the whole introduction to make our contributions clear. Actually “cumulative notifications of RTAs in force” and “cumulative number of RTAs in force” represent “signed” and “ratified” respectively according to WTO website.

8: You adopt the term RTA throughout, but I think you are actually talking about preferential trade agreements (PTAs) not simply regional trade agreements (confusingly the WTO uses the RTA term, but I would still clarify this if you continue to use RTA). This also provides an entry to speak more to the fact that PTAs are very heterogenous, some cover a few products only (thinking of some middle eastern agreements), whereas some cover most or all of the trade between signatories.

**Response:** Again, we very much appreciate your kind work. Actually we are also confused because the WTO uses the RTA term. However, we look it up on WTO website carefully and it writes: “RTAs are reciprocal preferential trade agreements between two or more partners”. We agree with you on this and we change the RTA term into PTA in this study.

9: In table 2 I would write out what the different fixed effects terms rather than simply giving them their Greek symbols.

**Response:** Again, we very much appreciate your kind work. We take you advice and change the Greek symbols into different fixed effects terms in our empirical tables.

We think each of your suggestions deeply and they are really helpful for our paper revision. Hopefully, we have clarified our contributions and linked it to existing literature. In this new version, we have significantly improve the language of the entire manuscript, and believe this new version is more readable.
