# Supplementary material for: The impact of preferential trade agreements on bilateral trade: A structural gravity model analysis
Source: PLoS One. 2021 Mar 30;16(3):e0249118. doi: 10.1371/journal.pone.0249118 (PMC8009400; doi:10.1371/journal.pone.0249118)
Supplement: Attachment — Submitted filename: Response to Reviewers.docx [file pone.0249118.s004.docx]

**Reply to Reviewers Comments for Paper PONE-D-20-21416** **R2**

**“The impact of Preferential Trade Agreements on Bilateral Trade: A Structural Gravity Model Analysis”**

**Dear Editor and Reviewers,**

We would like to commence by thanking the editor and the reviewers for their valuable time and constructive comments. Their expert knowledge of the field has helped us to strengthen the manuscript significantly. We very much appreciate Reviewer#3’s valuable suggestions, which is really helpful for us to revise the manuscript. We endeavored to address all the comments and our reflections are given below point by point. We have taken English service to make our paper more readable.

To help researchers benefit from our new measure, we upload our measure to the Journal website.

Sincerely,

The Authors

**Response to Reviewer #3’s Comments**

**Reviewer #3:**

**Reviewers' general comments:**

Overall, I think the manuscript is improved over the first version. In particular, the argument and writing is easier to follow on the whole. Moreover, I can see the paper being of interest to readers.

**Response:** Thank you very much for your valuable suggestions. We are grateful to your valuable suggestions, which is helpful for us to revise the manuscript. In this new version, we have taken English service to improve the language of the entire manuscript, and taken your advice to adjust the empirical section. Hopefully we can meet your demand and help you understand our contributions to existing literature. Again, thank you very much for your valuable suggestions.

**Specific comments:**

1: the abstract doesn’t do a great job motivating the study. In fact, it begins with two somewhat disputed claims (that PTAs increase trade and that structural gravity models are the best way to study). I would change this so that you are motiving the study around a debate/question or a contribution. So, for instance you could say “trade agreements are thought to raise trade-integration…… but existing measures are limited…” or something to this effect. Or alternatively you could just start by directly stating what your paper contributes. I would read papers with good abstracts to see what the best ones look like.

**Response:** Thank you for the valuable comments and suggestions. We take your advice and revise the abstract as follows:

Trade agreements are thought to raise trade integration, but existing preferential trade agreements (PTAs) are insufficient in measuring market access of products. This study develops a product-based coverage index of PTAs using the World Trade Organization (WTO) preferential trade agreements and calculates bilateral trade measures using the EORA multi-regional input-output (MRIO) tables covering 189 countries worldwide over the period 1990–2015; the structural gravity model is employed to test how PTAs affect bilateral trade. Our findings show that countries sharing a common PTA could boost the trade volume compared to those without PTAs, supporting the trade creation effect. However, the trade promotion effect of the product-based coverage index of PTAs is significant only if the member countries are low-and middle-income countries. Further, the wide range of product liberalization brought by PTAs can promote global production networks by stimulating the trade of intermediate goods. Our results are important for understanding the market access effect of PTAs with the increasing development of trade integration and global value chains (GVCs).

2: Make sure to change articles (a, an, and the) from definite to indefinite when the identity of the noun is unknow. For example, in the first sentence it should be “we develop a product-based coverage index.” rather than “we develop the product-based coverage index…”.

**Response:** Thank you very much for your careful reading and valuable comments and suggestions. We read the whole carefully and take English service to smooth the whole paper this time.

3: I am not sure that motivating the global value chain (GVC) aspect of the study solely with respect to COVID-19 is the right way to go (you aren't testing anything related to COVID-19). Yes, the pandemic makes it more important potentially, but I think it is an important dynamic to study even without the pandemic, as GVCs have increased in importance in recent years.

**Response:** Thank you for the valuable comments and suggestions. We motivate the global value chain without referring to COVID-19 in this version.

4: You still regularly do not spell out acronyms the first time you use them and/or you spell them out multiple times later, which you don’t need to do. For instance, EORA is used in the abstract without defining, and ISIC is used this way also. But, in other parts, you regularly spell out PTAs each time it is used.

**Response:** Thank you very much for your careful reading and valuable comments and suggestions. We read the whole carefully and take English service to smooth the whole paper this time. We try to spell out acronyms the first time we use them and use them after that. Sorry for our careless about this.

5: You do a better job in several places selling your product-based coverage measure, but the writing is still somewhat indirect. Make sure to state very clearly that previous models don’t have a fine-grained measure of market access (unless they do, and you should make this clear). This is one of the selling points of the paper and the prim value added. Explain very directly why we need this and what it adds to the literature on trade agreements!

**Response:** Again, we very much appreciate your kind work. The product-based coverage measure is one of our contribution to the related literature, and we stress the reason why we construct this measure in multiple places, especially in the literature review section. Existing literature referring to depth of PTAs focus on provisions beyond tariff, such as services, investment, standards, intellectual property, and competition rules, as well as a host of issues not directly related to trading, such as the environment. Some scholars measure the market access level brought by PTAs, while they mainly focus on service trade and calculate the index on service industry level. We consider commodities and service together and construct the product-based coverage index. We further investigate the trade effect of the product-based coverage index of PTAs. The literature review are as follows:

2 Literature review

In this section, we review the related literature to clarify our contribution from the literature perspective. We mainly focus on two strands of literature: the effect of PTAs on bilateral trade and the design of PTAs.

PTAs have been proliferating for the last twenty years. A large body of literature has studied various aspects of this phenomenon and study its design. Some researchers have highlighted that bilateral trade agreements are obstacles to free trade [34]. Regarding the obstacle [35–39], researchers argued that PTAs undermine multilateral growth and unleash a protectionist spiral. They further stated that PTAs effects like the spaghetti bowl effect and reduced countries’ incentive to enter multilateral trade agreements. Regionalism leads to welfare losses in both member and excluded countries [40]. In a Ministerial Meeting of the WTO in Doha (2001), governments expressed their opinion that “regional trade agreements can play an important role in promoting the liberalization and expansion of trade” [41]. The motivation for this evolution in PTAs is to ease tariff barriers and ‘new age’ matters such as foreign direct investment, services, labor, and environmental standards [28]. More scholars have applied the gravity model to estimate the effect of PTAs and find a positive effect on bilateral trade [39, 40]. However, most studies conceptualize PTAs as a dichotomous variable, namely whether countries sign an agreement or not and hence treat PTAs as if they were all equal in purpose while estimating the effects of PTAs. The results are fruitful, and they conclude that, on average, PTAs can promote bilateral trade for member countries. Some scholars estimate that the long-run effect of PTAs on bilateral trade flows is 100% and the effect varies considerably across trade agreements [42, 43]. Others applied the structural gravity model to analyze the PTAs and concluded that the average treatment effect of PTAs on trade flows was 236% [44]. Dembatapitiya and Weerahewa [45] investigated the SAFTA, European Union (EU), ASEAN, BIMSTEC, and North American Free Trade Agreement (NAFTA) regional trade impact and found mixed results. For example, the co-efficient for the EU is significant, while SAFTA, ASEAN, BIMSTEC and NAFTA do not significantly impact bilateral trade. Sampson [46] studied on the evolution of China’s PTAs and stated that the increasing network of China’s PTAs is important and strategic for the Asian region. Pant and Paul [47] evaluated PTAs for India and argued that PTAs are good for the intra-regional trade volume and welfare of countries. Scholars also assess the ex-post trade effects by applying the gravity model [48, 49]. Carre`re [50] studied ex-post PTAs and claimed that intra-regional trade mostly tied to a reduction in imports. However, regional agreements back weak governments to implement reforms and stabilize them despite domestic opposition. Partners may learn the advantages of liberalization once they practice limited free trade.

Until recently, scholars investigated the content and design of PTAs [51] and studied regional specifications [52] or explained functional differences in design, for example, dispute settlement [53] and flexibility provisions [54]. Furthermore, they found that tariff cuts, and other market access and trade-related provisions in PTAs concerning topics such as investments and intellectual property rights matter for trade flows [5], and that such deep agreements are usually flexible in adjusting their policies for other purposes and withdraw the PTAs without violating the terms of an agreement [6]. In addition to market access to goods, many PTAs today include provisions in trade disciplines such as services, investment, standards, intellectual property, and competition rules, as well as a host of issues not directly related to trading, such as the environment. At the beginning of the study of PTAs’ design, scholars usually focus on specific PTAs and measure the strength of a wide variety of provisions in the legal texts of PTAs [10, 55]. With increasing number of documents on PTAs available on the WTO website, scholars have extended their objects and started to pay greater attention to the scope and depth of these agreements [5–10,55–58]. They focus on the broader economic integration rights in goods, services, and factor markets brought by PTAs. For example, Dür et al. [5] used two different measures to operationalize the depth of PTAs. The first measure of depth is an additive index that combines seven key provisions included in PTAs, and the second one relies on latent trait analysis. Baccini et al. [6] measured the flexibility of PTAs and found a positive relationship between depth and flexibility for PTAs. Hofmann et al. [7] offered a detailed assessment of preferential arrangement. They examined the coverage and legal enforceability of provisions regulating a large set of policy areas. In addition to the depth of PTAs, many scholars have studied the breadth of PTAs [59–61]. Miroudot et al. [10] attempted to construct an industry-based coverage index of PTAs; however, they only studied 56 services PTAs. Limão claims that a broader PTA is one where partners seek to increase market access in product markets, and in markets for capital, labor, and technology [59]. Based on this, Hofmann et al. construct the measure based on a count of the provisions covered by a PTA and can focus either on the 18 ‘core’ provisions [60] and recent scholars also build a measure following this method [61]. Although some scholars have constructed variables to measure the heterogeneity of PTAs, most of them focus on the non-trade aspects of PTAs, and few measure the coverage index from the product-based perspective, which is important in the context of global production network.

In addition to the effect of PTAs on bilateral trade and the design of PTAs, this study also investigates how PTAs affect intermediate goods trade. Existing literature referring to the GVCs tends to study how deep trade agreements affect different trade types [61–63]. Laget et al. [62] use trade data in both value-added and gross forms to provide a complete picture of GVCs trade as possible and found that the positive impact of deep trade agreements on GVCs integration is driven by value-added trade in intermediate rather than final goods and services. In contrast to Laget et al. [62], scholars [61, 63] use the EORA multi-regional input–output (MRIO) data to derive variables for trade in value-added, as these offer greater country coverage. They found that broader PTAs have a larger impact on trade flows involving intermediates relative to flows involving all products, suggesting that GVCs trade is particularly sensitive to the scope of trade policy cooperation [61]. However, they measure the coverage index of PTAs based on the number of provisions, and we still do not know whether the products mentioned in the PTAs promote cooperation in the global production network. With the development of global production network, a product-based measure for the coverage of PTAs is needed to study the heterogeneity of PTAs and how this affects bilateral trade structure.

6: The example of your PTA_ratio variable and Chinese PTAs are good and illustrative. Two minor things though: 1) not sure if Iceland is one of the two most important non-EU countries (Britain, Norway,?); and 2) you have some pejorative sounding statements. For example, you say “mutually beneficial and win-win free trade agreements” when describing China’s PTAs -- try to keep language more neutral. Also, slightly confusing when you pivot from talking about China-Switzerland to China-Georgia.

**Response:** Again, we very much appreciate your kind work. In last version, we try to list as many PTAs signed by China as possible to verify our measure, and the results fit the facts well. In this version, we keep the discussion of China- Switzerland and China-Iceland and delete the China-Georgia to make paper more readable. And the revision is as follows:

We take China as an example to verify our product-based coverage index of PTAs , which is shown in Fig 2. China's regional trade agreements since 2000 show the characteristics of differentiation: the China-Switzerland free trade agreement and China-Iceland free trade agreement, which came into effect on 1 July, 2014, have a broad coverage in terms of trade in goods and trade in services. As important non-EU countries, Switzerland and Iceland are China’s important economic and trade partners in Europe. The two agreements have wide coverage, high level of openness, and many preferential policies. They are high-quality and wide-ranging free trade agreements. They are also one of the highest level and most comprehensive free trade agreements reached by China in recent years. The product-based coverage index shows that the coverage rate of goods and service trade of China-Switzerland free trade agreement is 100% and nearly 90% respectively, which has a strong fit with the official data.

Fig 2 The product-based coverage index of PTAs referring to China

7: Table 1. I am not sure why you put this here and not perhaps before table 4 where it is more applicable (but in general its location is not as important as explaining why you did it). Also, I am not sure why we need to know the correlation between the PTA dummy and the other 3 variables (you are correlating a binary variable and continuous variables)? Second, you state that multicollinearity is not a problem but your correlation coefficient between PTA ratio and flexscape is .65. This is pretty high. Better to check the variance inflation factor (VIF) between your independent variables and then, if the VIF is too high, take corrective measures. Also, of note, is that the correlation between you PTA ratio and depth is only .327. I would discuss this. I think it potentially makes your paper stronger by showing that you are measuring something that depth doesn’t capture.

In the paragraph below table 3 you note that you have included “other variables to measure the heterogeneity of PTAs” and then say that you included depth and flexibility in model 1. But this is very confusing: 1) model 1 in table 3 does not include your product-based coverage measure, and 2) model 1 doesn’t seem to include the other depth variables (are they estimated but not included in table 3). This also makes the correlations you presented in table 1 more confusing. What is the purpose now? In general, much more attention to detail needs to be taken in walking through all this. The results become confusing unless you are very careful, and this then makes the reader more and more skeptical.

**Response:** Again, we very much appreciate your kind work. In this version, we make two distinct adjustment about this. First,

In our baseline model, we do not include the depth and flexibility measure, because we want to show the basic effect of PTAs on bilateral trade with the factors controlled following most of existing studies. We include the depth and flexibility measure in the robustness checks to show that the results are robust taking other measure of PTAs into account. Before the robustness checks, we take your advice and put the correlation matrix table before table 4 (we delete the dummy variable in the correlation matrix table). Second, we check the variance inflation factor (VIF) and the values are all below 10, suggesting that there is no multicollinearity problem. The revisions are as follows:

*4.1 Baseline results of* *how PTAs affects bilateral trade*

First, we test the effects of PTAs on bilateral trade. The results of the panel structural gravity model are presented in Table 2. We first used the dummy variable (PTAs) to measure whether a trade agreement exists the two-countries, and the results are shown in column (1)–(3). The results in column (1) of Table 2 shows that the impact of PTAs on bilateral trade is positive and significant. This implies that a trade agreement helps increase the trade flow between the member countries of PTAs. These results are consistent with the existing study [75]. We also control for the economic scale effect. The economic scale effect (income-effect) of exporter/importer countries (agreement partner) on bilateral trade flow is significantly positive. Surely economic scale effect raises the bilateral trade between partners. It is remarkable to note that the magnitude impact (0.31 for importers, 0.35 for exporters) of both partners is significant at the 1% level but slightly different. This implies that trade will increase at a significant level on both sides which ultimately increases the welfare gains for both countries. The positive impact of income was validated with Yao et al. [76]. However, geographical distance negatively influences bilateral trade flow, consistent with the traditional gravity model results. This implies that distance causes a decrease in bilateral trade due to the shipping cost of transferring goods from one country to another. Therefore, adjacent countries promote bilateral trade rather than distant countries. Consistently, our results confirmed that the adjacent countries contribute positively to increasing bilateral trade, as the coefficient (0.3975) impact is significantly positive. Communication is important for promoting trade between two countries [77]. For example, each country has its rules and regulations, which are settled between the two countries through communication. Therefore, a common language can be a powerful tool to deal with these issues, that is, more communication in the same language, more understating more international supply chain and thereby more trade [76]. The colonial relationship also affects trade between two countries [77]. Our results also show the positive influence of colonial on trade follow. This means that the colonies strengthen the trade relationship. Currency helps to determine a country’s economic health. The same currency would help promote trade between countries as trade becomes cheaper if trading partners have the same currency [78]. In addition, our results showed a positive impact on trade flow.

To control for factors that may disturb the estimation, we added year-fixed effects and country-fixed effects. In column (2) of Table 2, we added country-pair fixed effects to control for all potential factors between countries. In this regression, the impact of PTAs on bilateral trade is again positive and significant at 1 % level. However, we found that the marginal effect of PTA on bilateral trade dropped from 0.165^[1]^ (as shown in column 1) to 0.038 (as shown in column 2), which means that when we control for more paired country factors, the marginal effect decreases. In addition, we found no change in the influence of exporter/importer countries income on bilateral trade flow as the coefficient impact is positive.

We further added the country-year fixed effects to control for the country-year level factors, which may disturb the estimation. The result in column (3), shows that the marginal effect of PTA on bilateral trade decreases further to 0.0356 while it is still significant at the 1% level. The marginal effect given by the structural gravity model indicates that the bilateral trade would increase by 3.56% if countries have RTA between them. Additionally, we investigated the impact of PTAs with the same currency holding other parameters constant. Results showed that PTAs increase bilateral trade flow if these countries have the same currency. Furthermore, a common currency is good for decreasing exchange rate volatility to zero [79, 80].

For the first three columns, we used the PTA dummies listed in the WTO. However, Baier et al. stated that dummy variables hardly capture the heterogeneous effect of PTAs [70]; hence, the marginal effect was not accurate. Therefore, we constructed the coverage index of PTAs. The results of the coverage PTAs index reported in column (4)–(6) of Table 2. The results showed that the impact of the coverage index is positive and significant at the 1% level. This implies that under the global production network background, the product-based coverage of PTAs can promote bilateral trade significantly. Similarly, others parameters promote bilateral trade. We tested the coverage index of PTAs impact on bilateral trade with economic scale holding others parameters constant. The results again validate the promoting effect of the coverage index on bilateral trade. The findings in the column (4) indicated that the coverage index of PTAs has a positive impact on bilateral trade. The same currency promote trade between the member countries. In conclusion, the baseline results of the structural gravity model showed that the bilateral trade would increase if two countries have a common trade agreement and the trade promotion effect is positively affected by the product-based coverage of PTAs suggesting that market access openness of PTAs can significantly promote bilateral trade, supporting the trade creation effect of trade agreement.

Table 2.

Empirical results of how PTAs affects bilateral trade

| Variables | MD  （1） | MD  （2） | MD  （3） | MD  （4） | MD  （5） | MD  （6） |
| --- | --- | --- | --- | --- | --- | --- |
| PTA | 0.1527*** | 0.0375*** | 0.0350*** |  |  |  |
|  | (0.024) | (0.007) | (0.005) |  |  |  |
| PTA_Ratio |  |  |  | 0.5711*** | 0.0320** | 0.0413*** |
|  |  |  |  | (0.071) | (0.014) | (0.010) |
| Lnyi | 0.3111*** | 0.2946*** |  | 0.3133*** | 0.2941*** |  |
|  | (0.004) | (0.004) |  | (0.004) | (0.004) |  |
| Lnyj | 0.3585*** | 0.3498*** |  | 0.3607*** | 0.3493*** |  |
|  | (0.005) | (0.005) |  | (0.005) | (0.005) |  |
| LnDis | -0.9903*** |  |  | -0.9876*** |  |  |
|  | (0.017) |  |  | (0.017) |  |  |
| Contig | 0.3975*** |  |  | 0.4067*** |  |  |
|  | (0.091) |  |  | (0.091) |  |  |
| Comlang | 0.1230*** |  |  | 0.1195*** |  |  |
|  | (0.028) |  |  | (0.028) |  |  |
| Colony | 1.0761*** |  |  | 1.0821*** |  |  |
|  | (0.093) |  |  | (0.093) |  |  |
| Comcur | 2.5615*** | 0.0782*** | 0.0346*** | 2.5605*** | 0.0764*** | 0.0313*** |
|  | (0.147) | (0.010) | (0.009) | (0.147) | (0.010) | (0.009) |
| Year FE | Y | Y |  | Y | Y |  |
| Country FE | Y |  |  | Y |  |  |
| Country-pair FE |  | Y | Y |  | Y | Y |
| Country-year FE |  |  | Y |  |  | Y |
| Obs | 721,149 | 721,135 | 721,135 | 721,149 | 721,135 | 721,135 |
| R2 | 0.8273 | 0.9896 | 0.9965 | 0.8274 | 0.9896 | 0.9965 |

*Note:*  **, **, and *** represent significance at the 10%, 5%, and 1% levels, respectively. The t-statistic of the robust standard deviation of the estimated coefficients is given in parentheses. The standard deviation is clustered at the country-pair level. MD= model*

*4.2 Robustness checks*

Scholars have constructed other variables to measure the heterogeneity of PTAs according to the content of PTAs [22–24]. To distinguish our product-based coverage index of PTAs from their measures, we added these variables in the robustness checks. We first compared the product-based coverage index of PTAs with the depth and flexibility measures, and the results are shown in Table 3. We can conclude that the product-based coverage index of PTAs is positively correlated with the depth and flexibility of PTAs, with correlation coefficients of 0.327 and 0.65, respectively. The results show that there are differences between the product-based coverage index and the depth index (depth_rasch) in measuring the coverage of PTAs. The product-based coverage index mainly focuses on the products mentioned in the PTAs, while the depth index covers the seven key provisions. The correlation between the product-based coverage index and flexibility of PTAs (flexescape) is 0.65, suggesting that PTAs are more flexible if they cover more products. We checked the variance inflation factor (VIF) to make sure that there is no multicollinearity problem. The results in the last column of Table 3 shows that there is no multicollinearity problem with the VIF values of the three variables below 10.

**Table 3.**

Correlation matrix of different measures of PTAs

|  | PTA_ratio | depth_rasch | flexescape | VIF |
| --- | --- | --- | --- | --- |
| PTA_ratio | 1.000 |  |  | 2.35 |
| depth_rasch | 0.327*** | 1.000 |  | 2.12 |
| flexescape | 0.650*** | -0.093*** | 1.000 | 1.37 |

Note: We calculated the correlation matrix using Pearson’s method. *, **, and *** represent 10%, 5%, and 1% significance levels, respectively. We use the bilateral trade variable as the independent variable and three measures of PTAs as the dependent variable to calculate the VIF value, which is shown in the last column.

We then added these measures of PTAs in our robustness checks to test the effect of the product-based coverage index of PTAs on bilateral trade. The empirical results in column (1) of Table 4 show that the coverage index of PTAs has a significant positive effect on bilateral trade. Column (1) shows the robustness check of the baseline models, with the depth and flexibility measures of PTAs controlled. We also find that the depth of PTAs can promote bilateral trade, but the marginal effect (0.0316) is less than the coverage index (0.0450). The flexibility of PTAs negatively affects bilateral trade, because the PTAs are flexible to adjust their policies for other purposes without violating the terms of an agreement. They could not cut the tariff as promised because they could withdraw the PTAs. This is important for countries to design flexible PTAs. Flexible PTAs provide for legally accepted opt-outs without leading to a de jure breach of an agreement and encompass exit options, duration and renegotiation clauses, reservations, escape clauses, and withdrawal clauses, and hence help members to cooperate better [23]. The product-based coverage of PTAs differs them in focusing on the market access openness of PTAs instead of other aspects.

8: Table 4. Explain why you decided to do this? I find the endeavor interesting and worthwhile, but it comes across as a bit ad-hoc. Provide some rationale. Also, maybe you don’t want to call it “robustness” checks. I think really what you are doing is showing how your measure is different from and complements depth and flexibility, frame it as such.

**Response:** Again, we very much appreciate your kind work. In this version, we take you advice and frame table 4 as robustness checks.

9: Your note for table 5 just says “see note under Table 3. I don’t think the notes are or should be the same

**Response:** Again, we very much appreciate your kind work. In this version, we write notes for each table in detail.

1. The marginal effect is calculated by equation:（e^coeffecient^ -1）*100%
